# Wirkungen in der partizipativen Gesundheitsforschung: Eine Einordnung in die Diskurse zum *Forschungsimpact*

**DOI:** 10.1007/s00103-020-03268-8

**Published:** 2020-12-29

**Authors:** Theresa Allweiss, Tina Cook, Michael T. Wright

**Affiliations:** 1 Institut für Soziale Gesundheit, Katholische Hochschule für Sozialwesen Berlin (KHSB), Köpenicker Allee 39–57, Berlin, 10318 Deutschland; 2 Faculty of Liberal Arts, Education and Social Sciences, Liverpool Hope University, Hope Park, Taggart Avenue, Liverpool, L16 9JD Vereinigtes Königreich

**Keywords:** Forschungsimpact, Wirkungen, Partizipative Gesundheitsforschung, Gesundheitliche Chancengleichheit, Research impact, Impact, Participatory health research, Equal health opportunities

## Abstract

Seit einigen Jahren beschäftigt sich die akademische Welt verstärkt damit, welcher gesellschaftliche Nutzen von Forschung ausgeht und wie er erhoben und dargestellt werden kann. Zu der Wirkung von Forschung, dem sogenannten Forschungsimpact, werden verschiedene Diskussionen geführt – kontrovers besonders in Ländern, in denen Impact mittlerweile ein Faktor bei der Verteilung von Fördergeldern geworden ist. Partizipative Gesundheitsforschung ist ein Forschungsansatz, der diejenige in die Forschung einbindet, deren Arbeit oder Leben im Forschungsinteresse stehen. Mit diesem Ansatz soll auch außerhalb der akademischen Welt Wirkung erzielt werden, um Veränderungen zum gesundheitlichen Wohlergehen anzustoßen und die gesundheitliche Chancengleichheit in der Gesellschaft zu erhöhen.

Der Beitrag geht den Fragen nach, wie Forschungsimpact verstanden und erhoben werden kann und welchen Beitrag die Wirkungen der partizipativen Gesundheitsforschung zu gesundheitlicher Chancengleichheit leisten können. Er geht exemplarisch auf Aspekte von Impactdiskussionen im Vereinigten Königreich und in Deutschland ein und bildet dann den aktuellen Stand der fachlichen Auseinandersetzung mit dem Themenbereich ab. Schließlich beschreibt er die Wirkfaktoren der partizipativen Gesundheitsforschung, die zu einer Stärkung gesundheitlicher Chancengleichheit führen können.

Nicht nur im Bereich der partizipativen Gesundheitsforschung ermöglicht es die Diskussion über Forschungsimpact, die Nachhaltigkeit und den Wert von Forschung zu bewerten und zu stärken. Da die vielseitigen Wirkungen der partizipativen Gesundheitsforschung jedoch das Potenzial haben, gesundheitliche Ungleichheiten zu verringern, sollten sie entsprechend wahrgenommen und anerkannt werden.

## Abstract (en)

In recent years, the academic world has been increasingly concerned with the social benefits of research and how they can be identified and demonstrated. Various discussions are being held on the impact of research, which are especially controversial in those countries where impact has become an important factor in the distribution of research funding. For participatory health research, a form of research that prioritises the engagement of those whose work or lives are the topic of the research, achieving social just impact outside the academic world is fundamental to its goal to reduce health inequalities.

This article explores the questions of how research impact can be understood and collected and whether the impact of participatory health research can contribute to equal health opportunities. It addresses aspects of impact discussions in the United Kingdom and Germany exemplarily and presents the current state of the professional debate on the topic. Finally, it describes the drivers of participatory health research that can lead to a strengthening of equal health opportunities.

The discussion of research impact allows for assessing and strengthening the sustainability and value of research, not only in the field of participatory health research. The diverse impacts of participatory health research have, however, the potential to reduce health inequalities and need to be recognised as such.

## Einleitung

Vor einigen Jahrzehnten begannen Wissenschaftler*innen sich über die eigene gesellschaftliche Verantwortung als Forschende Gedanken zu machen. Dies warf Fragen nach dem Wert von Forschung für die Allgemeinheit – ihrem Impact – auf. Um den Nutzen von Forschung für die Gesellschaft besser zu verstehen, wird sich seitdem zunehmend mit Forschungsimpact beschäftigt [1–3]. Der Begriff Impact kann mit verschiedenen Worten ins Deutsche übersetzt werden, beispielsweise mit Wirkung, Auswirkung, Bedeutung, Nutzen, Effekt, Einfluss oder Folge. Greenhalgh et al. definieren Forschungsimpact als Nutzen, der über die Erzeugung wissenschaftlicher Erkenntnisse und Theoriebildung hinausgeht [2]. Gemeint sind in diesem Zusammenhang die ökonomischen, umweltbezogenen, kulturellen, sozialen und gesundheitlichen Effekte von Forschung auf gesamtgesellschaftlicher Ebene, wozu in den Gesundheitswissenschaften ebenfalls die Verringerung gesundheitlicher Ungleichheit gehört.

In der partizipativen Gesundheitsforschung (PGF) werden Personen, die von der Thematik der Forschung betroffen sind (z. B. Fachkräfte, Patient*innen, Adressat*innen von gesundheitsfördernden Maßnahmen) in den Forschungsprozess eingebunden. Sie gestalten das Forschungsvorhaben aktiv mit, indem sie sich an den Entscheidungen über die Forschungsfragen, die Art der Datenerhebung, die Interpretation der Ergebnisse und die Verbreitung der Ergebnisse beteiligen. Die PGF möchte nicht nur Gesundheitsprobleme und ihre Ursachen beschreiben und erklären, sondern auch den notwendigen sozialen Wandel zur Verbesserung der Situation herbeiführen [4]. Die Ziele der PGF sind es, einerseits neue Erkenntnisse zu generieren und andererseits Veränderungen zur Förderung von Gesundheit und Wohlbefinden anzustoßen sowie gesundheitliche Chancengleichheit zu stärken [5].

Innerhalb der PGF hat sich ein Verständnis von Impact entwickelt, das sehr breit gefasst ist. So werden unter dem Begriff Forschungsimpact nicht nur beabsichtigte und unbeabsichtigte Veränderungen auf Makroebene (z. B. Gesellschaft, nationale Politik), sondern auch auf Meso- und Mikroebene (z. B. Kommunen, Institutionen und Individuen) subsumiert. Im Gegensatz zur Definition von Impact in der Evaluations- und Interventionsforschung steht Impact im Rahmen der PGF sowohl für kurzfristige als auch langfristige Wirkungen. Die International Collaboration for Participatory Health Research (ICPHR) definiert Forschungsimpact wie folgt ([6], Übers. d. Verf.):

Unter dem Begriff „Impact“ sind die zahlreichen Veränderungen vereint, welche die an der Forschung beteiligten Personen betreffen, sowie jene, die sich innerhalb des komplexen sozioökologischen Systems oder systemübergreifend in den Bereichen ereignen, in denen partizipative Gesundheitsforschung durchgeführt wird. Impact ereignet sich während des gesamten Forschungsprozesses und setzt sich nach dessen Abschluss weiter fort.

Die Anerkennung dessen, was die Entstehung von Wirkungen beeinflusst und wie verschiedene Faktoren dazu beitragen, ermöglicht es zu verstehen, wie sich Forschungsprozesse und -ergebnisse auf gesundheitliche Chancengleichheit auswirken können. Für die PGF, der das Streben nach Veränderungen innewohnt, ist es folgerichtig, Veränderungsprozesse zu analysieren und zu reflektieren. Zudem ist die Beantwortung der Frage, wie Partizipation als Katalysator für Veränderung wirkt, für sie von besonderer Bedeutung.

„PartKommPlus – Forschungsverbund für gesunde Kommunen“ ist ein partizipativ arbeitender Verbund, der sich das Ziel setzte, den eigenen Forschungsimpact samt seiner Entstehungswege zu erfassen, zu beschreiben und online^1^ zu veröffentlichen [7]. Um eine passende Vorgehensweise für dieses Ziel zu finden, wurde die britische Forscherin Tina Cook, welche Expertise in PGF und Forschungsimpact besitzt [8], als Prozessberaterin hinzugezogen und ein internes Grundlagenpapier verfasst. Der vorliegende Artikel basiert sowohl auf diesem Grundlagenpapier als auch auf dem binationalen Austausch über Wirkungen. Der Beitrag bildet Diskussionen ab, wie sie im Vereinigten Königreich und in Deutschland über Impact geführt werden, und erörtert, wie Forschungsimpact innerhalb und außerhalb der PGF verstanden, erhoben und bewertet wird. Es werden zudem Wirkfaktoren beschrieben, die zu einer Stärkung gesundheitlicher Chancengleichheit führen können.

## Diskurse

Die Gründe, die aus internationalem Blickwinkel zu einer intensiveren Beschäftigung mit Impact geführt haben, sind vielfältig. Neben ethisch-moralischen Motiven haben auch Erkenntnisinteresse, Verantwortungsgefühl sowie der Wunsch nach Qualitäts- und Effektivitätssteigerung dazu beigetragen [9, 10]. Generell ermöglicht die Auseinandersetzung mit Impact, die Nachhaltigkeit von Forschungsvorhaben zu bewerten und zu stärken. Damit wird teilweise der politischen Forderung nach einem verbesserten Forschungs-Praxis-Transfer und einer aktiven Wissenschaftskommunikation Rechnung getragen [11]. Die Bemühungen von Hochschulen, ihre gesellschaftliche Verpflichtung („third mission“) zu erfüllen, sind in den letzten Jahren gestiegen und verdeutlichen die Relevanz des Themas [12]. Auch soll gegenüber verschiedenen Interessengruppen, Geldgeber*innen bzw. Steuerzahler*innen Rechenschaft bezüglich Wert, Effizienz und Effektivität der von ihnen in Auftrag gegebenen und/oder finanzierten Forschung abgelegt werden [10]. International wird zunehmend versucht, anhand von Forschungsimpact die Mittelvergabe möglichst effektiv zu gestalten [13] und die begrenzten Ressourcen so zu verwenden, dass der Nutzen maximiert und sogenannter Forschungsabfall („research waste“; [10]) vermindert wird. Gerade in den Ländern, in denen Impact mittlerweile ein wichtiger Faktor bei der Verteilung von Fördergeldern ist, wird unter dem Begriff „Impactagenda“ kontrovers darüber debattiert.

### Beispiel Vereinigtes Königreich

Das Vereinigte Königreich besitzt eine relativ lange Geschichte der Beschäftigung mit Forschungsimpact. Es ist dort zu einer Notwendigkeit geworden, dass Forschung wirkungsvoll ist und dass Forschende in der Lage sind, die Wirkungen ihrer Arbeit zu artikulieren und nachzuweisen. Impact ist ein integraler Bestandteil von Ausschreibungen in der Gesundheits- und Sozialforschung geworden. Das National Institute of Health Research verlangt zum Beispiel, dass ein Plan für öffentliches Engagement und Wirkung als Teil des Antrags vorliegt [14]. Der Economic and Social Research Council erwartet von Antragsteller*innen, dass sie die Wirkungen ihrer Forschung beschreiben und dabei bestimmen, ob es sich um „akademische Wirkungen“ (Bedeutung für die Wissenschaft) und/oder wirtschaftliche und gesellschaftliche Wirkungen handelt [15].

Die Finanzierung von Forschungsaktivitäten erfolgt im Vereinigten Königreich durch Drittmittel und durch eine leistungsorientierte Mittelzuweisung an Universitäten. Das Research Excellence Framework (REF) ist das Bewertungssystem, mit dem die Regierung die Qualität und Güte der Forschung beurteilt, die in wissenschaftlichen Einrichtungen durchgeführt wird [16]. Seit 2014 fordert das REF narrative Fallstudien ein, in denen beschrieben werden soll, wie sich die durchgeführten Forschungsprojekte jenseits des akademischen Bereichs auswirkten. Die Ergebnisse des REF werden verwendet, um die Höhe der öffentlichen Forschungsgelder zu ermitteln, die jede britische Hochschule für den Zeitraum bis zum nächsten REF (in der Regel 5–7 Jahre) erhält. Der Betrag hängt davon ab, wie die jeweilige Forschung durch das REF eingestuft wird. Im REF 2014 wurden 25 % der Gesamtpunktzahl von dem beschriebenen Forschungsimpact abgeleitet. Im nächsten REF, das Ende 2021 fällig ist, sind es fast 30 % der Punktzahl.

Die Reaktionen von Forschenden auf die Einführung des REF 2014 waren unterschiedlich. Viele Akteur*innen der angewandten und partizipativen Forschung vermuteten, „ihr Moment an der Sonne sei endlich gekommen“ ([17], Übers. d. Verf.). Doch wurde die Art und Weise, wie Forschungskommissionen und Geldgeber*innen Forschungsimpact identifiziert und gemessen haben, in diesen Bereichen oft als unpassend angesehen [8, 18]. Alle Forschenden, egal welcher Richtung, waren aufgefordert, ihre Wirkungen so zu artikulieren, dass sie innerhalb der gesetzten Maßstäbe und quantitativ ausgerichteten Assessmentverfahren erkennbar wurden. Das führte zu dem Problem, dass partizipativ Forschende den Impact ihrer Arbeit nicht nur unterschätzten, sondern dass sie sogar verschiedene durch ihre Forschung hervorgerufene Wirkungen nicht erkennen konnten. Indem nur das artikuliert wurde, was gemeinhin als Wirkung anerkannt wurde, gerieten andere, alternative Formen der Wirkung aus dem Blickfeld [8]. Pain et al. [18] erklären dies damit, dass versucht wurde, Forschungsimpact „als ein konkretes, sichtbares, zeitlich und räumlich fixiertes Phänomen zu messen, das eine Partei einer anderen Partei zufügt … obgleich intensive Koproduktion ein Prozess ist, der oft eine graduelle, durchlässige und diffuse Reihe von gemeinsam getragenen Veränderungen einschließt“ ([18], Übers. d. Verf.).

Aber auch Forschende aus anderen Disziplinen waren beunruhigt über den Rahmen, der für die Bestimmung ihrer Wirkungen vorgegeben wurde. Fragen zum Nachweis der Signifikanz von Impact und seiner Entwicklung im Laufe der Zeit kamen auf und wurden beispielsweise von Vertreter*innen der Grundlagenforschung gestellt. Die Wissenschaftler*innen waren besorgt, dass sie die Wirkungen ihrer Forschung im Zeitrahmen eines geförderten Forschungsprojekts nicht vorhersehen oder erkennen könnten. Es wurde die Befürchtung geäußert, dass das REF Universitäten dazu verleiten könnte, Forschungsvorhaben, die Ziele innerhalb eines sehr spezifischen linearen Pfades messen können (z. B. biomedizinische Gesundheitsforschung), zu bevorzugen und langfristig andere wertvolle Forschung (z. B. aus den Sozialwissenschaften) zu benachteiligen [8].

Um die Wirkungen einer Forschungsinstitution für das REF darzustellen, werden Wissenschaftler*innen von ihrer Universität ausgewählt. Auswertungen der REF-Fallstudien aus 2014 zeigten, dass diese – unabhängig vom Geschlechterverhältnis der Akademiker*innen in den Fakultäten – in der Regel von älteren Männern eingereicht wurden [19]. Durch diese Verzerrung könnten sich zudem negative Auswirkungen auf die Karrierechancen von Frauen und Nachwuchsforscher*innen ergeben. Eine geschlechtersensible Form der Impacterfassung könnte indes dazu beitragen, bestehende Ungleichheiten zu reduzieren [20].

Die kritische Debatte im Vereinigten Königreich zum Thema Impact ist offen und dauert an. Das Forschungsnetzwerk UK Research and Innovation kündigte im Januar 2020 überraschend an, dass Förderanträge in Zukunft keinen spezifischen Abschnitt mehr enthalten sollen, in dem die Antragstellenden Wirkungspläne im Voraus festlegen müssen. Das zeigt, dass sich Verfahren und Einstellungen ändern und die Impactagenda einen fließenden und formbaren Charakter hat [21].

### Diskussion in Deutschland

Eine eigenständige fachbereichsübergreifende Debatte über Forschungsimpact gibt es im deutschsprachigen Raum bisher (noch) nicht. Beiträge zu diesem Thema behandeln häufig entweder nur Wirkungen innerhalb der Wissenschaft und arbeiten sich an metrischen Indikatoren wie dem Impactfaktor von Wissenschaftsjournalen ab [22–24] oder sie sind eine Reaktion auf internationale Entwicklungen wie das REF [13]. Die auch in Populärmedien verbreiteten Meinungen geben überwiegend Befürchtungen wieder, etwa, dass durch eine Impactagenda die Freiheit der Forschung verloren ginge und durch eine Fokussierung auf kurzfristigen Nutzen einige Wissenschaftsbereiche, zum Beispiel die Grundlagenforschung oder die Geisteswissenschaften, benachteiligt würden [25, 26]. So warnte etwa 2016 Strohschneider, der ehemalige Präsident der Deutschen Forschungsgemeinschaft: „Der im Imperativ des ‚Impact!‘ geronnene, zeitlich wie sachlich verkürzte Instrumentalismus eines ökonomistischen Forschungsdiskurses rüttelt in ähnlicher Weise an den Pfeilern einer pluralistischen Gesellschaft und Wissenschaft wie die vielfältig grassierenden Populismen mit ihrer manifesten Aversion gegen Expertise und Reflexivität“ [27]. Dabei werden Systeme zur Erfassung von Impact vor allem dann abgelehnt, wenn diese die Mittelvergabe beeinflussen. Der deutsche Wissenschaftsrat sieht beispielsweise die in Antragsverfahren im Voraus zu tätigende Einschätzung von Wirkungen kritisch und warnt vor der Orientierung an kurzfristigen Nutzenerwartungen [28]. Gleichzeitig befürwortet er aber eine differenzierte Auseinandersetzung mit Forschungsimpact – insbesondere um zu klären, unter welchen Bedingungen Forschung Wirkung entfalten kann [28]. Auch das REF-Verfahren wird nicht nur skeptisch betrachtet. Einige Wissenschaftler*innen können sich eine Adaption für das deutsche Wissenschaftssystem gut vorstellen, da es die gängige Praxis der rein auf Publikationen beruhenden Impactmessung hin zu einem Wirkungswege beschreibenden Fallstudienansatz wandelt [22, 29].

Es ist jedoch schwierig zu lokalisieren, wer wie über Impact spricht, da andere Themen, wie Wissenschaftskommunikation und Transfer, die eng mit Wirkungen verknüpft sind, aktuell besondere Aufmerksamkeit erfahren [11]. In Deutschland scheint es vor allem die transdisziplinäre Forschung zu sein, die eigene Akzente im Bereich Forschungsimpact setzt [30]. Innerhalb der PGF übernimmt derzeit PartKommPlus eine Vorreiterrolle, indem sich der Verbund verstärkt mit den Wirkungen partizipativer Forschung beschäftigt [7, 31].

Es ist in Deutschland bislang nicht zwingend notwendig, sich intensiv mit Forschungsimpact und seinen Erfassungsmöglichkeiten zu beschäftigen, da es kein verpflichtendes Assessmentverfahren wie das REF gibt. Allerdings sehen sich Forschende hierzulande bei der Bewerbung um Fördergelder immer häufiger damit konfrontiert, Aussagen zu dem erwarteten gesellschaftlichen Impact des Vorhabens zu formulieren – insbesondere bei internationalen Förderprogrammen. Somit kann davon ausgegangen werden, dass Impact zunehmend sowohl den Intellekt als auch die Gemüter hiesiger Forscher*innen anregen wird.

## Kategorisierung und Entstehung von Forschungsimpact

Forschungsimpact kann sich in verschiedenen Bereichen zeigen und differenziert eingeteilt werden. Beispiele für typische Wirkungskategorien in der PGF finden sich in Tab. 1. Der Blick richtet sich hier gezielt auch auf die Mikroebene, mit dem Bewusstsein, dass auf Veränderungen in diesen Kategorien weitere „größere“ Wirkungen aufbauen können – beispielsweise wenn Menschen durch die Mitarbeit an Forschungsprojekten Kompetenzen erweitern und sich gestärkt fühlen, um ihre Lebens- oder Arbeitssituation zu verbessern. Wirkungen auf Personengruppen und Beziehungen sind in der PGF essenziell, da durch den Aufbau von Ressourcen und nachhaltigen Netzwerken die Anliegen von weniger gut organisierten Gruppen, die besonders von gesundheitlicher Ungleichheit betroffen sind, besser vertreten werden können. Die Einteilung in Wirkungskategorien kann bei der Erhebung von Forschungsimpact hilfreich sein und es Forschenden erleichtern, ihre Arbeit zu reflektieren. Allerdings gelingt eine klare Aufteilung nicht immer, da Wirkungen auf verschiedene Ebenen ausstrahlen, sie sich gegenseitig bedingen und sich abhängig von Zeitpunkt und Perspektive verändern können (siehe Infobox 1).

**Table 1:** 

Wirkungskategorie	Unterkategorien (Erklärung/Beispiele)
An der Forschung beteiligte Individuen	An der Forschung beteiligte Menschen, die direkt von der Forschungsthematik betroffen, aber nicht unbedingt wissenschaftlich ausgebildet sind (z. B. Fachkräfte, Patient*innen, Adressat*innen von gesundheitsfördernden Maßnahmen) An der Forschung beteiligte Menschen, die wissenschaftlich ausgebildet, aber nicht unbedingt direkt von der Forschungsthematik betroffen sind Andere Mitwirkende (z. B. Interviewpartner*innen, Proband*innen)
Beziehungen	Beziehungen, Partnerschaften, Netzwerke zwischen Beteiligten der Forschung und darüber hinaus
Personengruppen	Gruppen und breitere Bevölkerung (z. B. Ziel‑/Adressat*innengruppen, Klient*innen/Patient*innen, Gemeinschaften, Gemeinden)
Forschung	Forschungsagenda, Forschungsprozess, Forschungsethik
Handlung	Praxis (z. B. in der Gesundheitsförderung, Behandlung oder Pflege)
Strukturen	Organisationen und Strukturen (z. B. Leistungserbringer, soziale Träger, Verwaltungen)
Politik	Politik (auf unterschiedlichen Ebenen)

Die Entstehung von Impact und der Bereich der Wirkungsmechanismen scheinen bisher noch nicht so gut untersucht zu sein wie die verschiedenen Wirkungskategorien [32]. Untersuchungen zu Wirkungswegen zeigten, dass eine Vielzahl an unterschiedlichen Wegen zu Wirkungen führen kann [32, 33]. Gerade partizipativ forschende Wissenschaftler*innen beschreiben die Entstehung von Wirkungen als komplex, nichtlinear, dynamisch, wellenhaft oder verwoben [18, 34].

## Impactmodelle

In den letzten Jahren wurden Modelle und Rahmenkonzepte entwickelt, um Impact besser erklär- und/oder erhebbar zu machen [2]. Da in der Wissenschaftslandschaft diverse Auffassungen über Forschungsimpact bestehen, haben sich sehr unterschiedliche Ansätze entwickelt. Während einige Modelle vor allem Wirkungskategorien (und passende Indikatoren) vorgeben, bilden andere auch Wirkungswege ab [10, 35]. Häufig finden sich in der Literatur lineare Modelle, bei denen Impact das Ergebnis eines geradlinigen Ablaufs ist und die teilweise einer Geber-Empfänger-Logik folgen (z. B. Input → Aktivität → Output → Outcome → Impact). Beispielsweise wird angenommen, dass Wirkungen über die Dissemination von Publikationen angestoßen werden, was nicht nur von partizipativ Forschenden mehrheitlich als zu vereinfachend abgelehnt wird, da sich Forschungsimpact eher selten und nur unter begünstigenden Umständen direkt infolge von akademischen Veröffentlichungen einstellt [32]. Innerhalb der PGF und angrenzender Forschungsrichtungen entstanden daher eigene nichtlineare Konzeptionierungsvorschläge [36].

Ein Beispiel ist das Community-Based Participatory Research (CBPR) Conceptual Model [37]. Es ist in 4 Bereiche untergliedert (Kontext, Partnerschaft, Handlung und Forschung, Wirkung) und zeigt mögliche Entstehungswege von Wirkungen auf [38]. Es unterscheidet zwischen mittel- und langfristigen Wirkungen: Mittelfristige Wirkungen beinhalten beispielsweise die Kompetenzentwicklung bei den beteiligten Personen und Institutionen, Veränderungen in Richtlinien und Verfahren oder den Aufbau von dauerhaften und gleichberechtigten Kooperationen; die langfristigen Wirkungen beruhen auf mittelfristigen Wirkungen und zeigen sich unter anderem in Form von gesellschaftlichen Transformationen, Zugewinn an sozialer Gerechtigkeit oder erhöhter gesundheitlicher Chancengleichheit [37]. Eine aktuelle Metaanalyse von 100 Übersichtsarbeiten zu partizipativen Forschungsansätzen unterstützt mit ihren Ergebnissen den Aufbau des Modells und untermauert die Relevanz des Zusammenspiels der 4 Hauptbereiche für das Erzielen von gesundheitlicher Chancengleichheit [39]. Auf Basis des CBPR-Konzeptmodells und unter Berücksichtigung der Prinzipien der Gesundheitsverträglichkeitsprüfung (Health Impact Assessment) wurde überdies ein Rahmenkonzept zur Evaluation der Wirksamkeit partnerschaftlicher Zusammenarbeit hinsichtlich der Verringerung von gesundheitlicher Ungleichheit entwickelt [40].

Die ICPHR betont, dass Modelle dieser Art nur dann ihre Nützlichkeit entfalten, wenn sie zum lokalen Forschungskontext und den dort gelebten Werten passen sowie offen für Diversifizierung und unerwartete Ergebnisse sind [6]. Auch kann es unter Umständen hilfreich sein, statt eines umfassenden Rahmenkonzepts, mehrere an die Gegebenheiten im Projekt angepasste Ansätze zu kombinieren [36]. Forschende sollten sich also aus den bestehenden Ansätzen den oder die passendsten für ihre Arbeit heraussuchen bzw. diese an ihren eigenen Kontext anpassen [10].

## Herausforderungen bei der Erfassung von Forschungsimpact

Die Erfassung von Forschungsimpact ist anspruchsvoll und birgt einige Schwierigkeiten. Ein grundlegendes Problem ist, dass Kausalitäten zwischen den meisten Forschungsvorhaben und deren Wirkungen schwer nachweisbar sind. Wirkungen können nur selten eindeutig bestimmten Forscher*innen, Projekten oder Institutionen zugeschrieben werden, da sie oft im Zusammenspiel verschiedener Faktoren, auch außerhalb des Forschungsumfeldes, entstehen und nicht unbedingt aus einer einzigen Forschungsarbeit resultieren [9, 10]. Ein Beispiel dafür ist das langsame unterschwellige Einschleichen von wissenschaftlichen Erkenntnissen („knowledge creep“; [41]) in das Bewusstsein von Entscheidungsträger*innen, das Beschlüsse beeinflussen kann, ohne dass sich die Akteur*innen darüber unbedingt bewusst wären. Es kann daher manchmal schwierig sein, aussagekräftige Belege und Hinweise für Wirkungen zusammenzutragen.

Da Wirkungen dynamisch sind und sich mit der Zeit weiterentwickeln, fallen sie je nach Zeitpunkt der Erhebung unterschiedlich aus [9]. Die Zeitspanne zwischen Forschung und Forschungsimpact variiert dabei stark. Laut einer Studie benötigt Forschung zu Herz-Kreislauf-Erkrankungen beispielsweise zwischen 10 und 25 Jahren, bis sie Behandlung und Prävention beeinflusst [42]. Selten wird ein Forschungsprojekt so lange finanziert, dass es über mehrere Jahre hinweg seinen Impact erheben könnte. Der Zeitfaktor betrifft ebenso die Bewertung von Impact, da sich auch die Einschätzung des Nutzens im Laufe der Zeit verändern kann [9].

Metrische Indikatoren, wie gesundheitliche oder ökonomische Kennzahlen, werden als aussagekräftige und eindeutige Formen der Evidenz angesehen [9, 32]. Dementsprechend werden sie bei der Erfassung von Forschungsimpact häufig eingesetzt. Besonders beliebt ist die Messung von Veröffentlichungen, Zitierungen und Beiträgen in angesehenen Wissenschaftsjournalen [10]. Dieses Vorgehen ist jedoch in die Kritik geraten, da solche bibliometrischen Kennzahlen in erster Linie die Verbreitung oder Vermarktung von Forschungsergebnissen abbilden und wenig über die Wirkungen auf gesellschaftlicher Ebene aussagen [23]. Die alleinige Verwendung von Metriken kann den Blick auf vorherbestimmte Wirkungskategorien einengen. Für bestimmte Wissenschaftsbereiche, wie die Sozial- oder Geisteswissenschaften, sind diese Ansätze zudem ungünstig, da sich ihre Wirkungen allein mit quantitativen Indikatoren schwer abzeichnen lassen [32]. Problematisch ist auch, dass Metriken dazu benutzt werden könnten, Forschungsimpact in Geldwert umzurechnen, etwa um die Kosten eines neuen Theaters mit dem eines Krankenhauses zu vergleichen [9].

## Methodisches Vorgehen bei der Erfassung von Forschungsimpact

Es gibt keinen Konsens darüber, wie Forschungsimpact und dessen Entstehungswege erfasst und dargestellt werden sollen. Die Wahl der entsprechenden Methoden und Indikatoren hängt stark von dem zu untersuchenden Forschungsprojekt ab. Da sich Wirkungen sehr divers präsentieren können, wird oft mit multimethodischen Einzelfallstudien gearbeitet [2]. Es wird dazu geraten, möglichst vielfältige Daten, beispielsweise aus Umfragen, Interviews, Fokusgruppen, Projektdokumentationen, Beschreibungen oder qualitativen Feldnotizen, als Hinweise oder Belege für Impact zu erheben [10].

Methoden, die zudem besonders für die Erfassung von Wirkungen in der PGF geeignet erscheinen, sind das „community asset mapping“ und die soziale Netzwerkanalyse [43]. Beim Community Asset Mapping geht es darum, die materiellen und immateriellen Ressourcen einer Bevölkerungsgruppe abzubilden, bei der sozialen Netzwerkanalyse um die Erhebung der Beziehungen innerhalb (und außerhalb) der Forschungsgemeinschaft [44]. Um eine Veränderung im Verlauf feststellen zu können, sind hierbei mindestens 2 Messzeitpunkte notwendig. Da sich Wirkungen auf verschiedenen Ebenen zeigen, sie intendiert oder nichtintendiert sein können und auch von unterschiedlichen Beteiligten verschieden bewertet werden können, ist eine offene Herangehensweise passend [44]. Forschungsimpact sollte darüber hinaus nicht nur von einer Beteiligtengruppe identifiziert werden. In der PGF wird Impact als das Ergebnis eines gemeinsamen Reflexionsprozesses angesehen, in dem sich alle Beteiligten aus ihrer Perspektive darüber verständigen, was als Wirkung gilt [34]. Auf diese Weise kann vermieden werden, dass durch eine zu große Abhängigkeit von professionellen Perspektiven entscheidende Wirkungsdetails auf lokaler Ebene übersehen werden.

## Forschungsimpact und gesundheitliche Chancengleichheit

Unter dem Begriff gesundheitliche Chancengleichheit wird die Zielvorstellung verstanden, dass alle Menschen die gleichen Chancen haben sollen, um gesund sein und bleiben zu können [45]. Diese Zielvorstellung ist so relevant, da Gesundheitschancen in der Gesellschaft, abhängig von Merkmalen wie Bildung, Einkommen oder Geschlecht, Alter und Migrationshintergrund, sehr unterschiedlich ausfallen. Um zu verstehen, wie gesundheitliche Ungleichheit entsteht, wird oft verwiesen auf soziale Ungleichheit sowie Unterschiede in Lebensbedingungen, gesundheitsrelevantem Verhalten und dem Gesundheitszustand [46]. Aktuell wird außerdem die Bedeutung von sozialen Netzwerken hervorgehoben [47]. In den Gesundheitswissenschaften wird davon ausgegangen, dass „Maßnahmen zur Verringerung gesundheitlicher Ungleichheiten nur dann erfolgreich sein können, wenn die aktive Einbindung derer gewährleistet ist, deren Gesundheitszustand verbessert werden soll. Ohne ihre Partizipation auf möglichst allen Ebenen der Planung und Entscheidungsfindung ist eine erfolgreiche Durchführung kaum möglich“ [46].

Die PGF arbeitet mit Gruppen, die besonders stark von gesundheitlicher Ungleichheit betroffen sind, und bindet gleichzeitig weitere für den Forschungsgegenstand relevante Akteur*innen ein. Es wird Entscheidungsmacht mit diesen Gruppen geteilt. Auch wird der Forschungsimpact von denjenigen mitbestimmt, für die die Forschung Veränderungen bewirken soll. Das ist wichtig, da die Einflussmöglichkeiten auch hierbei oft ungleich verteilt sind, was dazu führen kann, dass Entscheidungen über die Relevanz von Forschung und deren Wirkungen von Fachleuten getroffen werden, die nicht immer die Bedürfnisse der Menschen verstehen, deren Gesundheit gefördert werden soll. Diejenigen, die selten beteiligt sind und selten gehört werden, haben die Möglichkeit, ihre Stimme zu erheben und mit ihrer Stimme etwas zu bewirken. Dabei werden Wirkungen von allen Beteiligten durch deren Zusammenarbeit bedingt, nicht allein durch das Handeln der Wissenschaftler*innen oder die Dissemination von Ergebnissen. Diese Arbeitsweise stärkt als Schlüsselfaktor für die Generierung von Impact die Partizipation und das Empowerment der beteiligten Personen und Gruppen. Die kommunikativen Prozesse in der PGF ermöglichen die Koproduktion von Wissen und den Aufbau von nachhaltigen Netzwerken. Die Wirkungen, die so auf individueller, gemeinschaftlicher und struktureller Ebene durch PGF angeregt werden, können, wie das CBPR-Konzeptmodell veranschaulicht, auf Makroebene ausstrahlen und einen Beitrag zu gesundheitlicher Chancengleichheit leisten.

## Fazit

- Forschende bewerten die zunehmende Fokussierung von Forschungsimpact sehr unterschiedlich. Neben der Befürchtung, dass eine Impactagenda die Wissenschaft zu stark eingrenzen könnte, werden auch das Potenzial für gesellschaftliche Innovationen und eine Neubewertung der unterschiedlichen Forschungsansätze gesehen.
- Die Konzeptualisierung und die Theoriebildung in Bezug auf Forschungsimpact stehen noch am Anfang und es ist abzusehen, dass sich diese in den nächsten Jahren weiterentwickeln werden. Innerhalb der PGF umfasst das Verständnis von Forschungsimpact einen breiten und integrativen Ansatz zur Identifizierung und Erfassung von beabsichtigten wie unbeabsichtigten Wirkungen. Die PGF löst Grenzen zwischen wissenschaftlichem Wissen und Erfahrungswissen auf, verändert Machtverhältnisse und stellt auch Annahmen über die Form und Funktion von Forschungsimpact infrage. So verändert die PGF, wer Entscheidungen darüber trifft, was Wirkungen sind bzw. was als Wirkung zählt. Der Forschungsansatz besitzt das Potenzial, direkten Einfluss auf gesundheitliche Ungleichheiten zu nehmen, welche von den Betroffenen auf lokaler Ebene als wichtig erachtet werden.
- Das Interesse an dem Thema Forschungsimpact ist angesichts des Schwerpunkts auf Aktion und Veränderung für die PGF eine Chance. Die systematische Erfassung und Darstellung der Veränderungen, die durch partizipative Forschungsprozesse ausgelöst werden, können von wesentlicher Bedeutung für die Weiterentwicklung und Wirksamkeit des Forschungsansatzes sein. Gesundheitliche Chancengleichheit kann mit einem besseren Verständnis von Wirkungswegen in Forschung und Praxis gezielter adressiert werden. Dafür sind ein umfassendes Verständnis von Forschungsimpact und die Anerkennung von unterschiedlichen Wirkungsformen und deren Entstehungswegen notwendig.

### Infobox 1 Eigenschaften von Wirkungen in der partizipativen Gesundheitsforschung. Eigene Zusammenstellung. Quellen: [8, 18, 34, 43, 44]

- Wirkungen entwickeln sich über verschiedene Zeiträume hinweg (sie entstehen gemeinsam im Forschungsprozess und nach dessen Abschluss),
- können sich gegenseitig beeinflussen und aufeinander aufbauen (Wirkungen können Wellen schlagen),
- entstehen häufig nichtlinear,
- können intendiert oder nichtintendiert sein,
- können positiv oder negativ bewertet werden, wobei je nach Zeitpunkt und Perspektive die Beurteilung unterschiedlich ausfallen kann,
- sind kontextsensitiv,
- zeigen sich in verschiedenen Bereichen und auf verschiedenen Ebenen (Makro‑, Meso- und Mikroebene),
- können individuell, gruppenbezogen oder strukturell sein.

## Footnotes

1 Siehe: www.partkommplus.de.
